# Strengthening implementation of catch-up vaccination and multi-disease screening for new migrants

**DOI:** 10.1093/eurpub/ckac129.115

**Published:** 2022-10-25

**Authors:** S Hargreaves

**Affiliations:** The Migrant Health Research Group, St Georges, University of London, London, UK

## Abstract

Dr Hargreaves will discuss the current guidance for EU/EEA countries on public health considerations for newly arrived migrants, with a specific focus on catch-up vaccination delivery across the life course in mobile populations. She will explore current initiatives and best practices to ensure child, adolescent and adult refugees and migrants are included in catch-up planning and delivery for missed vaccines and missed doses and to align them with the host country vaccine schedule.

